# The musculoskeletal consequences of latissmus dorsi breast reconstruction in women following mastectomy for breast cancer

**DOI:** 10.1371/journal.pone.0202859

**Published:** 2018-08-28

**Authors:** Nicole E. Blackburn, Joseph G. Mc Veigh, Eilis M. Mc Caughan, Richard D. Kennedy, Stuart A. McIntosh, Iseult M. Wilson

**Affiliations:** 1 Centre for Public Health, School of Medicine, Dentistry and Biomedical Sciences, Queen’s University Belfast, Belfast, United Kingdom; 2 UKCRC Centre of Excellence for Public Health, Belfast, United Kingdom; 3 School of Clinical Therapies, College of Medicine and Health, University College Cork, Cork, Ireland; 4 Institute of Nursing and Health Research, Ulster University, Coleraine, United Kingdom; 5 School of Medicine, Dentistry and Biomedical Sciences, Queen’s University Belfast, Belfast, United Kingdom; 6 Centre for Health and Rehabilitation Technologies, Institute of Nursing and Health Research, Ulster University, Jordanstown, United Kingdom; University of Bristol School of Social and Community Medicine, UNITED KINGDOM

## Abstract

**Introduction:**

Current evidence suggests that patients who have latissimus dorsi (LD) breast reconstruction following mastectomy for breast cancer can experience long-term shoulder dysfunction. However, as there is no standardised assessment or follow-up period within the literature, findings are conflicting. This research aimed to investigate the impact on daily living of immediate and delayed LD breast reconstruction in women following mastectomy for breast cancer.

**Methods:**

Both qualitative and quantitative methods of enquiry were used. A focus group study explored the musculoskeletal consequences of surgery as perceived by the women (n = 15) and their healthcare professionals (n = 11). A questionnaire survey was administered (n = 159), including a range of outcome measures to quantify both the physical and psychosocial impact of LD breast reconstruction. Dyad interviews were also conducted in order to determine the impact of surgery on function and activities of daily living (ADL) from the woman’s perspective and that of her significant other (n = 8).

**Results:**

The qualitative studies highlighted a lack of preparedness and unrealistic expectations regarding functional recovery among women and their significant others’. Post-surgery it was apparent that women weighed up reduced shoulder function against survival, demonstrating resilience in their approach to coping with this adaptive way of living. The survey identified low to moderate effect on the outcomes assessed (n = 159), however, node removal significantly impacted certain aspects of quality of life (p<0.05) and disability (p = 0.04).

**Conclusions:**

Breast reconstruction using the LD had an impact on shoulder function and some ADL, which impacted not only on the women but also family and significant others. Despite the functional implications associated with surgery, findings would suggest that shoulder dysfunction is not their main concern. This work identified that women and their significant other require further information to clarify expectation regarding recovery, highlighting the changing priorities of women throughout their journey from diagnosis into long-term recovery.

## Introduction

The primary aim of breast cancer treatment is to remove the tumour and administer any adjuvant treatment necessary [1]. Surgery is usually the initial stage of treatment, either by lumpectomy, wide excision or mastectomy, all of which can alter body image and change breast aesthetics greatly [2, 3]. The type of surgery prescribed will depend on a number of factors including; the size of the tumour, and whether or not the cancer has spread outside of the breast [4, 5]. In some cases various options may be offered, including; breast conserving surgery (lumpectomy, wide local excision, quadrantectomy) or mastectomy [6]. A number of side effects of surgery can occur, including; limited range of movement (ROM), seroma formation, numbness and tingling, scarring, pain, lymphoedema, body image concerns, trauma and fatigue [6]. Surgical treatment may also include axillary nodal dissection, either as sentinel lymph node biopsy or an axillary node clearance. These procedures may also have an impact on complications such as, shoulder morbidity, seroma formation, lymphoedema and altered sensation [7].

Breast reconstruction following mastectomy has been considered an important stage in the rehabilitation of breast cancer, and the UK National Institute for Health and Clinical Excellence (NICE) recommends that reconstruction should be available to all women with breast cancer at the initial surgery [8]. Breast reconstruction can be performed at the same time as initial mastectomy (immediate) or at a later date when the patient has fully recovered from initial surgery (delayed). Autologous tissue transfer is a popular approach to breast reconstruction [9, 10]. In autologous tissue reconstruction, a piece of tissue known as a flap; containing fat, blood vessels, skin and at times muscle, is taken from somewhere else in the body and used in the reconstruction of the breast [3, 11]. Functional sequeale, such as weakness and dysfunction rarely develop in the autologous transfers that do not involve muscle [9, 12].

Latissimus dorsi (LD) breast reconstruction has traditionally been viewed as an effective method of autologous breast reconstruction, with shoulder impairment reportedly being minimal and having insignificant functional consequences [13, 14]. However, there is disagreement within the literature regarding the outcomes associated with LD breast reconstruction. While the procedure has demonstrated positive aesthetic outcomes, with apparent minimal donor-site morbidity [15–20], commonly reported complications include seroma formation, wound infection, flap necrosis and shoulder dysfunction [21, 22]. Studies have shown that objective measurements of shoulder range, power, strength and endurance have decreased following the loss of the LD [17, 23–27].

Previous literature exploring the effectiveness of LD reconstruction has mainly focused on body image, aesthetic results and wound healing [28, 29] with little in-depth investigation of the impact of this surgery on shoulder function and how this may affect everyday living. Some literature has explored musculoskeletal outcomes and recent research suggests that shoulder function is significantly impaired years after surgery [17, 30] and that breast reconstruction using LD flap may result in long-term upper limb musculoskeletal dysfunction [26].

The extent and impact of the musculoskeletal consequences of breast reconstruction on functional mobility and activities of daily living (ADL) have yet to be fully understood, therefore, further investigation is required. This study aimed to explore the impact of surgery on shoulder function and ADL as perceived by the patients and healthcare professionals and also to identify the extent of musculoskeletal problems associated with this surgery and the impact these problems may have on a patient’s life and that of their family and friends.

## Methods

Appropriate ethical and research governance approvals (13/NI/0141 and 15/NI/0183) were obtained from the Office for Research Ethics Committee Northern Ireland (ORECNI). In order to provide deeper insights and greater understanding of the complexities of the functional effects of LD breast reconstruction and the experiences of breast cancer survivors and their significant others, both qualitative and quantitative methodologies were used in this research. Consequently, focus groups, a questionnaire survey and dyad interviews were undertaken. The focus groups were conducted to explore the impact of LD breast reconstruction as perceived by the women and the healthcare professionals. Subsequently, a survey, using validated outcome measures, was administered to quantify the extent of the issues raised during the focus groups. Finally, dyad interviews were carried out to explore whether the impact of surgery extended beyond that of the women to her partner/significant other. The methods were selected to answer different research questions about the same phenononon providing a broader understanding of the topic being studied through triangulation, something that could not be achieved by a single method of investigation.

### Focus groups

The aim of this focus group study was to explore the extent of musculoskeletal problems associated with LD breast reconstruction and their impact on function and ADL, as perceived by the women themselves and the healthcare professionals who manage this client group. Women attending review clinics, who fulfilled the inclusion criteria, were informed of the study by the surgeon or the breast care nurse. Posters were displayed and leaflets were also available in each clinic and out-patient department to advertise the study. Any patients who demonstrated a potential interest in the study were also given an information sheet with further details regarding the research. Interested and willing participants were asked to consent to their contact details being passed to the research team. Potential participants were screened for inclusion to the study. Verbal information regarding the purpose of the focus group and the main areas for discussion were given to participants and verbal consent obtained; information regarding venue times and location was also given. Suitable and willing participants were invited for written formal consent and participation in the focus group.

Twenty six participants for the focus groups were recruited from three of the four main NHS centres for breast reconstruction, within the Northern Ireland Health and Social Care Trusts. Three focus groups were conducted with women with breast cancer and two with health care professionals. The healthcare professionals consisted of breast care nurses and physiotherapists working with women following LD breast reconstruction. Women were included if they met the following criteria: (1) had undergone uni-lateral LD breast reconstruction, following mastectomy for breast cancer, (2) were at least nine months post-operative to ensure completion of adjuvant therapies such as chemotherapy and radiotherapy, (3) had sufficient English language to understand and take part in a focus group discussion and (4) were over the age of 18. Eligible women attending review clinics were invited to join the study by their surgeon or the breast care nurse.

Before the focus group, each of the women in the women’s groups was asked to complete a brief, two-page questionnaire that included demographic information such as age, diagnosis, staging of breast cancer, cancer treatments (surgery, chemotherapy, radiotherapy), type of reconstruction (immediate or delayed), and time lines (diagnosis, mastectomy, reconstruction). During the focus groups, an exploratory questioning approach was used. This allowed for quality analysis and enhanced consistency [31]. The questions used in the focus group topic guide were developed by the steering group with advice from a health service user, and were also informed by the available literature (see S1 File). After each focus group there was a debriefing between the facilitator and the moderator, to highlight any key themes or contrasting ideas, and reflect on the discussions in relation to the topic guide. All focus group discussions and field notes were transcribed in full, verbatim, with any identifiable information anonymised. Descriptive summaries were also written and circulated to each participant who was then asked to verify the accuracy of the descriptive summary [32]. Once all participants had responded to the descriptive summaries and verified that they were accurate accounts of the discussion, transcripts were then coded and analysed. The transcript data were independently coded, and subsequently the codes and categories were agreed by consensus, following a thematic approach [33]. These were then analysed in order to determine the emerging themes. The themes were identified and sorted independently by individual researchers in the first instance, and then by consensus for secondary, more in-depth analysis [34].

### Questionnaire survey

In order to obtain a more comprehensive picture of the extent and impact of the musculoskeletal dysfunction in women following LD breast reconstruction, it was deemed appropriate to conduct a large scale survey, in order to provide an adequate representation of the extent of the functional impact of LD flap surgery on these patients. All eligible women who underwent LD breast reconstruction in Northern Ireland between 2000 and 2015 were invited to take part in the study. The aim of this survey was to quantify the extent of the musculoskeletal problems associated with LD breast reconstruction, and the impact that these problems may have on the woman’s life.

An anonymous, postal, questionnaire-survey was used to gather detailed and personal information from women aged 18 years and above, who had undergone breast reconstruction using the LD, following mastectomy for breast cancer. Based on the findings from the focus group study, a number of validated tools were selected in order to further explore the impact of surgery on relevant outcomes including, pain [35], quality of life (QoL) [36] and function [37]. As a result, the survey consisted of five validated questionnaires: The Brief Pain Inventory (BPI), Fear Avoidance Belief Questionnaire (FABQ), The EuroQol EQ-5D, The Disabilities of Arm, Shoulder and Hand (DASH) Questionnaire and the Satisfaction with Life Domains Scale for Breast Cancer (SLDS-BC). Additionally some questions were included in the questionnaire based on (i) findings from the focus group study, (ii) current knowledge regarding surgery and its secondary consequences in people with breast cancer, and (iii) expert opinion. All women aged over 18, who underwent LD breast reconstruction following mastectomy for breast cancer, in Northern Ireland between 2000 and 2015, were included in the study. Recruitment took place over 12 months across the four NHS Trusts in Northern Ireland providing breast reconstruction surgery. Theatre Management Systems in each Trust were accessed to identify women who had undergone LD flap reconstruction. Cross-checking with Patient Administration Systems minimised the risk of communicating with families of deceased patients, which would have been likely to cause distress.

Participants were identified by the designated principal investigator (PI) at each site. Each PI reviewed the database within their Trust to confirm eligibility. Women identified by the PI were informed of the study and sent an individually coded survey pack by post. Each questionnaire had a unique identification number on the cover which corresponded with each patient number on the database, held by the PI. Non-responders were sent a reminder letter three weeks after the first mailing. Completion (part or all) and return of the questionnaire was considered as giving full informed consent, unless the respondent specifically withdrew consent. Women could withdraw at any time by contacting the researcher, returning a blank questionnaire, or by not responding to the second invitation.

Data were entered onto the SPSS database, (version 22), and cleaned and anonymised prior to analysis. In addition, 10% of the questionnaires were independently cross-checked by another member of the research team for accuracy. Prior to carrying out statistical analysis, the data were assessed for normality of the distribution of scores using Kolmogorov-Smirnov, with a non-significant result (p≥0.05) indicating a normal distribution. In addition, the original means and 5% trimmed means were compared visually in order to investigate whether any extreme scores were having a strong influence on the mean. Data analysis included descriptive statistics, with simple frequency counts, means and standard deviations (SD). A paired sample t-test was carried out to determine the difference in ability ‘today’ compared with ‘before’ LD breast reconstruction. The Mann-Whitney U Test was used as the non-parametric assessment to test for differences between two independent groups on a continuous measure and the Independent t-test was used as the parametric alternative. Sub-group analysis was carried out between a number of independent groups, including: laterality, type of procedure i.e. (immediate vs delayed), whether or not surgery included node removal and whether or not they were prescribed hormonal therapy. Differences in the outcome measure scores were compared across groups using the Kruskal-Wallis Test and One Way ANOVA for non-parametric and parametric assessments respectively. Differences were compared between groups dependent on: age at diagnosis, time since surgery and staging of breast cancer. In order to compare between groups, age at diagnosis and time since surgery were divided into three relatively equal groups in order to make for fair comparison of scores [38], as recommended by a statistician.

### Semi-structured in-depth interviews

The overall aim of this study was to determine the impact of breast reconstruction surgery specifically using the LD muscle, on function and ADL from both the woman’s perspective and their identified ‘significant other’. Individual experiences were explored and perceptions of the impact of breast reconstruction on shoulder function and ADL were investigated, in order to gain an in-depth insight into the lived experience of those affected [39]. Questions were open and focused, in order to encourage respondents to speak freely and openly about their own perceptions of the topic, while maintaining some control over the content and process of the interviews [40] (see S2 File). In-depth interviews were conducted with a number of dyads, i.e. two individuals: the woman who had undergone breast reconstruction surgery using the LD muscle, and a significant other, who was identified by the woman. Recruitment for the dyads was carried out in two phases: firstly, the woman was recruited and then the significant other. Women were recruited from charities and special interest groups within Northern Ireland. Eligibility for the women in the interview study was the same as that for the focus group study, with the inclusion that their elected significant other also participated. If the potential participant met the inclusion criteria and consented in principle to being involved in the study, she was then asked to nominate the family member or friend (significant other) who was most involved or impacted upon during her experience. The significant other had to be over 18 and involved with the woman pre-operatively and post-surgery (close relationship, co-habitant, etc.). An information sheet was given to all women and their elected significant others, including further details of the study and contact details of the research team in order to allow them to make a fully informed decision regarding their participation in the research. Suitable and willing participants were sent out a consent form to complete individually, along with a stamped addressed envelope to facilitate return. All women and their significant other’s provided written informed consent.

Within the dyad interviews, the woman and her elected significant other were interviewed separately, in order to ensure that their individual perceptions were captured and to reduce the risk of one participant influencing another’s response to a question. Interviews were held in a location convenient for participants (such as the individual’s home, different University campuses or charities’ facilities). Interviews for each dyad was conducted one after the other or on different days depending on participant availability. Prior to beginning the interview discussion, each woman was asked to complete a brief, one-page questionnaire, including demographic and treatment information. The data from the dyad interviews were transcribed in full, verbatim. All data were anonymised and the two samples (women and significant others’) were analysed separately. The transcript data were independently coded, and subsequently the codes were agreed by consensus within the research team. These codes were then analysed in order to identify the emerging themes and sub-themes, initially by individual researchers, and then agreed by consensus for secondary, more in-depth analysis. In order to ensure rigour, the research team adopted a number of strategies including validation of the data by the interviewees [41]. Descriptive summaries were written and circulated to each participant who was then asked to comment on the accuracy of the account. By doing so, we hoped to accurately and credibly reflect the respondents’ interpretation of responses.

## Results

### Focus group findings

Fifteen women (n = 5, n = 7, n = 3) and 11 healthcare professionals (n = 6, n = 5) attended one of the five focus groups. The duration of the focus groups ranged from 74 to 97 minutes. The healthcare professionals’ focus groups included breast care nurses (n = 8) and physiotherapists (n = 3) who had experience of working with women who had LD breast reconstruction (range: 2–26 years). All participants contributed to all discussions.

Women reported common complications associated with LD flap breast reconstruction, including: lymphoedema, seroma formation and problems with wound healing. Functional difficulties experienced by the women included: tightness in the shoulder and back, cramping, weakness in grip, pain and discomfort at the donor site, reduced power, inability to carry heavy weights and numbness. Many women also emphasised the struggle with personal care (e.g. washing behind their back), ADL (e.g. house work) and leisure activities (e.g. swimming). In addition to the common functional challenges described above, several discrete yet notable challenges emerged from the discussions, for example, some women expressed concerns with balance and increased levels of muscle fatigue when doing activities that involved the operated side. Despite the small sample size, these symptoms illustrate the potential complexity of the management challenges women undergoing this surgery may face. Characteristics of the women are presented in Table 1.

**Table 1:** Characteristics of women (n = 15).

Characteristic	Number	%
*Age (years)*		
Mean (SD)	56 (7.5)	
Range	45–71	
*Type of Reconstruction*		
Immediate	12	80
Delayed	3	20
*Method of Reconstruction*		
LD	4	27
LD + Implant	11	73
*Operated Side*		
Dominant	6	40
Non-Dominant	9	60
Time since Reconstruction (months)		
Mean (SD)	62 (28.9)	
Range	12–122	

Three themes were identified from the women’s focus group data in relation to the impact of LD breast reconstruction on their daily living. These were (i) preparation and awareness (ii) coping, and (iii) self-management. The over-arching theme to emerge from the women’s data was resilience. Analysis of the data from the healthcare professionals revealed the distinct roles of the physiotherapists and breast care nurses and highlighted different beliefs regarding LD flap recovery timelines among the healthcare professionals. Patient contact varied, with pathways differing between the Trusts. Three themes were identified in relation to the impact of LD breast reconstruction as perceived by the healthcare professionals, including (i) the healthcare professionals’ perceptions of their role in supporting patients undergoing LD flap surgery (ii) the healthcare professionals’ perceptions of the physical implications associated with surgery, and (iii) inconsistencies within service provision.

It was evident that reduced strength and restricted function of the shoulder was not a main concern for patients and in most cases, despite not being informed of these potential limitations prior to surgery, they accepted it as a consequence of the procedure. Patients demonstrated self-management in their approach to coping with life following LD flap surgery. They demonstrated a responsibility for their own aftercare and revealed different coping strategies in order to deal with the challenges of recovery. Patients recognised the need for support and their dependency on others, specifically their identified significant other, in the immediate postoperative period and for some women the need for support was prolonged due to the lasting effects of treatment and LD flap surgery.

### Survey findings

A total of 244 surveys were distributed and an overall response rate of 65% was achieved (n = 159), comparable with that reported elsewhere in the literature [42]. Full demographic and medical information is presented in Table 2.

**Table 2:** Survey respondents demographic and medical information.

Demographic and Medical Information (n = 159)	N	%
**Staging of Breast Cancer**	**146**	
Stage 0	11	7.5
Stage I	7	4.8
Stage II	46	31.5
Stage III	36	24.7
Stage IV	4	2.7
Don’t Know	42	28.8
**Treatment Received**	**159**	
Chemotherapy	106	66.7
Radiotherapy	83	52.2
Hormone Replacement Therapy	20	12.6
Targeted Biological Therapy	20	12.6
Hormone Therapy	11	6.9
**Current Cancer Treatments**	**74**	
Hormone Therapy	61	82.4
Chemotherapy	6	8.1
Biological Targeted Therapy	5	6.8
Radiotherapy	2	2.7
**Lymphoedema**	**159**	
Yes	26	16.4
No	132	83
Don’t Know	1	0.6
**Employment Status**	**156**	
Full-Time Paid Employment	92	59
Part-Time Paid Employment	28	17.6
Housewife	17	10.7
Self-Employed	8	5
Unemployed	5	3.1
Part-time Unpaid Employment/Volunteer	3	1.9
Active Retired	2	1.3
Retired for Medical Reasons	1	0.6

During the focus groups, women identified specific functional activities that were difficult following surgery. Survey respondents were asked to think about these tasks in terms of the level of difficulty they experienced carrying out the activity when ‘today’ was compared with ‘before surgery’. These measures were included to determine the transferability/generalisability of these problems from the small focus group population to the larger survey sample that was representative of 65% of the eligible women with LD flap surgery in Northern Ireland. A paired sample t-test was carried out to determine the difference in their ability to carry out the functional activities ‘before’ surgery compared with ‘today’. A significant difference was found for all activities (p<0.005), the results are presented in Table 3.

**Table 3:** Questionnaire survey (n = 159): non-validated outcome measures.

Functional activities ‘before’ compared with ‘today’*	N	Sig.
Reaching up to a high shelf	153	0.000
Dressing activities behind your back	154	0.004
Pushing down with your arm	155	0.001
Opening a tight jar	154	0.000

*Paired samples t-test

The results from the validated outcome measures demonstrated that women who had breast reconstruction using LD had low to moderate effect across all outcome measures. The results from the validated outcome measure scores are presented in Table 4.

**Table 4:** Questionnaire survey (n = 159): Validated outcome measure scores.

Outcome Measure	Mean (SD)	Possible Range	Effect
FABQ Physical Activity Subscale	11.0 (± 7.3)	0–24	Low
FABQ Work Subscale	8.4 (± 10.1)	0–42	Low
EuroQol Mobility	1.8 (± 0.96)	1–5	None—Slight
EuroQol Self-Care	1.7 (± 0.92)	1–5	None—Slight
EuroQol Usual Activities	2.2 (± 1.1)	1–5	Slight—Moderate
EuroQol Pain/Discomfort	2.5 (± 1.0)	1–5	Slight—Moderate
EuroQol Anxiety/Depression	2.0 (± 1.1)	1–5	Slight—Moderate
BPI Severity	3.4 (± 2.3)	0–10	Mild—Moderate
BPI Interference	3.5 (± 2.9)	0–10	Mild—Moderate
DASH	29.0 (± 23.9)	0–100	Moderate
SLDS-BC	79.9 (± 32.5)	27–189	Moderate

The findings from this study indicate that LD breast reconstruction has an impact on the functional ability of patients undergoing this specific procedure, with the results demonstrating low to moderate dysfunction among the group. Subgroup analysis suggested that those women who underwent axillary dissection had significantly worse disability scores (p = 0.04) as per DASH and worse QoL scores regarding mobility (p = 0.01) and self-care (p = 0.03) as per EuroQoL. The results from this subgroup analysis are presented in Table 5. Further subgroups analysis demonstrated there was no impact on outcome of arm dominance (dominant versus non-dominant), type of procedure (immediate versus delayed), current treatment received (hormone treatment vs none or other), time since reconstruction surgery (months), age at diagnosis (years) and staging of breast cancer (1–3).

**Table 5:** Subgroup analysis*: Node removal.

	Node Removal		Statistics			
	N (Yes)	N (No)	U	z	p	r
DASH	123	20	870.5	-2.09	0.04	0.18
FAQQ PA Subscale	118	18	760.5	-1.94	0.05	-0.17
FABQ Work Subscale	113	17	729.5	-1.64	0.10	-0.14
BPI Interference	118	17	757.5	-1.63	0.10	-0.14
SLDSBC	128	20	1179.5	-0.57	0.58	-0.05
EuroQol Mobility	127	21	896.5	-2.66	0.01	0.218
EuroQol Self-Care	127	21	987.5	-2.17	0.03	0.179
EuroQol Usual Activities	127	21	1237	-0.56	0.58	-0.05
EuroQol Pain/Discomfort	127	20	1046	-1.32	0.19	-0.11
EuroQol Anxiety/Depression	127	21	1072	-1.52	0.13	-0.13
EuroQol VAS (O-100)	125	21	1161.5	-0.84	0.40	-0.07

*Mann-Whitney U Test

These data suggest that undergoing axillary dissection may significantly impact function and QoL greater than LD flap surgery itself. However, due to the relatively small sample size within this subgroup it cannot be assumed that axillary dissection is an independent risk factor for worse disability, although the findings do merit further investigation.

### Dyad interview findings

Four dyads were recruited from charities and special interest groups within Northern Ireland. The duration of the interviews ranged from 37 minutes to almost 73 minutes in length. The dyad relationships included a civil partnership, two husband and wife couples and a sister-sister relationship. Characteristics of the women are presented in Table 6.

**Table 6:** Characteristics of women (n = 4).

Characteristic	Number	%
*Age (years)*		
Mean (SD)	36.8 (13.4)	
Range	24–58	
*Type of Reconstruction*		
Immediate	2	50
Delayed	2	50
*Method of Reconstruction*		
LD	3	75
LD + Implant	1	25
*Operated Side*		
Dominant	2	50
Non-Dominant	2	50
*Time since Reconstruction (months)*		
Mean (SD)	39.8 (34.6)	
Range	9–90	

Similar to the findings from the focus groups, analysis of the women’s dyad data revealed that despite women encountering various challenges as a result of surgery, they reflected positively on their overall experience. Three themes emerged from the women’s dyad data surrounding (i) significance of support, (ii) the relative importance of outcomes relating to surgery and (iii) a responsibility for their own aftercare. The results from this analysis reiterated the focus group findings surrounding the lack of follow-up concerning the musculoskeletal implications of surgery and the differences in the physiotherapy pathways between Trusts. Furthermore, these findings support the conclusions from the focus groups regarding how women view their shoulder function in terms of other aspects of recovery, such as; the aesthetic outcome of surgery and the psychological benefits associated with undergoing reconstruction surgery.

Three themes were identified in relation to the impact of LD breast reconstruction on function and ADL, as perceived by the significant others. These three themes were (i) lack of preparedness (ii) role adjustments, and (iii) impact on daily living. It was evident that while the women were dealing with the shock of the cancer diagnosis, their significant others dealt with the practicalities that stemmed from such diagnosis and the resultant treatment protocols. This led to behavioural changes occurring within the dyad which in turn had an impact on certain roles at home. The impact of surgery varied among the dyads; however a commonality among them all was the apparent change in dynamics in the immediate postoperative period. Behavioural changes occurred as a result of the functional impact of LD flap surgery, with certain behaviours at home needing to be modified in order to adapt to the situation. The significant others reported increased involvement in a number of caregiving activities, including; assisting with personal care, household chores, mobility, transportation, and emotional support. For those with young children, the caregiving role extended beyond the immediate postoperative period, with some significant others taking on additional roles for extensive durations.

The overarching theme to emerge from the women’s dyad data and that of the significant others was regarding managing expectations of surgery. The analysis of the dyads exposed a disparity between how they anticipated that the surgery would impact their function and ADL and the actual reality of the recovery process. It was apparent that both individuals within the dyad were unprepared for the postoperative pain, restricted movement and reduced strength in the initial weeks following surgery. The physical implications associated with surgery seemed to lessen in severity as the women went further into their recovery, with some dyads affected by the impact of surgery and treatment more so than others.

## Discussion

The aim of this research was to investigate the impact on daily living of LD breast reconstruction in women following mastectomy for breast cancer. The findings demonstrate that breast reconstruction using the LD may have an impact on shoulder function and some ADL, which impacts not only on the women but also family and significant others. The results highlight the complex issues that these women are facing as a result of their cancer diagnosis, their subsequent treatment protocols and the additional impact of LD breast reconstruction. Women recalled that during the immediate postoperative period following surgery, their main concerns were that the tumour had been removed and they were undergoing any necessary adjuvant treatment. As they further progressed into their recovery, concerns shifted from apprehensions about overcoming the cancer to the aesthetic outcome of the reconstruction and the functional limitations that were on-going since surgery.

Following a cancer diagnosis, survival is evidently the main focus of attention to the exclusion of everything else. However as time passes it would appear that issues that were not a major concern initially, may become more important [43, 44]. For instance, within the current study it was apparent that following diagnosis patients were under pressure to make a number of crucial decisions regarding their treatment options, all within a limited time period. Similarly, Gorman *et al*. [43] reported that young breast cancer survivors’ perspectives on treatment decisions and fertility were mainly motivated by survival and that it was not until later that other issues were raised. Furthermore, a recent study conducted by Javid *et al*. [44] explored what health-related quality of life (HRQL) domains and processes of care defined positive outcomes from both the patient and clinician perspective. The findings revealed that the domains most commonly prioritised as important across the entire surgical experience were emotional well-being, education/information, communication with the care team and the process of care. These findings support the current work, demonstrating the complexity of issues patients face preoperatively, and highlighting the importance of information and communication throughout the experience.

A common theme to emerge from both the focus group study and dyad interviews was the lack of preparedness regarding the musculoskeletal impact of LD flap surgery. Understanding and managing patients’ expectations can improve patient satisfaction [45]. Therefore, the importance of setting realistic expectations is essential for ensuring optimal patient care [46]. A comprehensive review conducted by Bodai and Tuso [47] evaluated long-term medical issues and lifestyle recommendations in breast cancer survivorship, highlighting the importance of patient education regarding the long-term sequelae of breast cancer and its treatment. ‘Being resilient’ was the main theme to emerge from the focus group data and was apparent within the dyad interview results. A review carried out by Molina *et al*. [48] assessed resilience during one or more stages of the cancer continuum. Their review was based on the approach that promoting resilience is a critical element of patient psychosocial care, and by optimising mechanisms of adaptation that these, in turn, can enable resilience among patients. This was evident within the current study whereby the women demonstrated a number of coping mechanisms, including adaptation which resulted in them reporting positively on the overall outcome of surgery, despite facing a number of challenges along the way.

A common finding within the literature, is the apparent elevated disability scores in the immediate postoperative period, with recovery timelines varying from three months [13] up to three years post LD flap [49]. It is also apparent that few, if any, participants recover to their pre-surgery state, however, in the majority of studies minimal long-term impairment is reported [11, 30–33]. In 2002, Hunsaker *et al*. [50] published normative data for the DASH outcome measure. The mean DASH score from the sample in the current study 29.0 (±23.9) is considerably higher than that of the normative mean DASH of 10.1 (±14.68). These results would suggest that LD breast reconstruction does impact on upper limb function and can result in shoulder disability.

The BPI findings from the current study are in accordance with previous literature published in the area [51, 52], suggesting that LD flap surgery does not result in the increased incidence of pain severity or interference with daily activity. The FABQ is commonly used to measure fear of movement and fear avoidance beliefs in patients with musculoskeletal conditions, including back and shoulder pain [53]. In the current study, low fear avoidance was reported among this patient group. It is likely that individuals dealing with a life and death situation, i.e. cancer diagnosis, will prioritise survival over any subsequent pain and or dysfunction, whereas those people who only have to deal with pain and or dysfunction [54, 55], without the life and death issue, will prioritise that and therefore as a result may report higher fear avoidance.

Spagnola *et al*. [56] administered the SLDS-BC questionnaire to assess QoL in adult female breast cancer patients (n = 153). The results from the current study demonstrated moderate satisfaction with life among the group, similar to that presented by Spagnola *et al*. [56]. This would suggest that despite women reporting mild to moderate dysfunction following LD flap, their satisfaction with life remains quite good. This could be an indication that in terms of the relative importance of outcomes regarding their recovery, shoulder function may not be one of their main concerns when considering they have recovered from their cancer diagnosis.

Subgroup analysis suggested that those women who had node removal during surgery had significantly worse disability as per DASH and QoL regarding mobility and self-care as per EuroQol. The findings therefore suggest that undergoing additional surgery, (node removal) may significantly impact function and QoL, more so than the LD flap surgery itself, or perhaps the impact may be as a result of a combination of node removal and LD flap surgery. This would indicate that the effects associated with having either sentinel and/or axillary nodes removed prior to surgery may have detrimental effects on certain outcomes relating to functional morbidity. Several studies have investigated the impact of LD breast reconstruction on QoL and shoulder function [57], however for a number of studies, assessing functional morbidity following LD flap surgery has only been a small part of the study objective [8, 58–59]. The current study is the first to implement a range of outcome measures specifically investigating the impact of this surgery on shoulder morbidity and ADL.

## Limitations

Regarding the focus group study, including an objective measure of shoulder ROM as well as strength, comparing the operated and non-operated side would have allowed the researcher to quantify the limitations and/or restrictions that resulted from LD flap surgery. Also, as identified within the focus group discussions, the surgeons have more contact, in most cases, with the patient than the physiotherapist. Therefore, it may have been beneficial to include a sample of surgeons in order to explore their perceptions of the issues discussed.

Due to the subjective nature of the survey there is the potential for recall bias, therefore this must be considered when interpreting the outcomes relating to their perception of their pre and post functional abilities. A limitation of the dyad interviews was regarding the small sample size (n = 8). Recruitment was maximised through the charities and special interest groups, however the sample size may have been improved if the researcher had recruited through research governance within the Trust.

## Implications for clinical practice

The findings presented within this study, demonstrate that this patient group has a number of challenges as a consequence of LD breast reconstruction which result in some enduring long-term implications. The current model for this patient group focuses on treatment of the disease with ongoing investigation to detect recurrence. However, Stout *et al*. [60] proposes a prospective surveillance model (PSM) which includes preoperative rehabilitation, evaluation and education; early postoperative rehabilitation, re-assessment and exercise prescription and ongoing surveillance. This PSM aims to identify changes and detect early signs of physical impairment in the hope of promoting early intervention to optimise recovery and return to premorbid levels of function. This framework could be adopted in the current system in order to ensure any physical symptoms that patients are enduring following surgery are identified and treated early, thus avoiding any unnecessary functional decline and promoting active recovery.

## Conclusions

In conclusion, there is evidence to suggest that LD breast reconstruction has an impact on shoulder function and certain ADL. This impact can extend beyond the woman, to her family, impacting roles and behaviours, dependent on individual circumstances and preparedness. This impact has greater bearing in the immediate postoperative period with the severity varying among patients. Women demonstrate resilience in how they cope with the after effects of surgery, promoting a positive message for this patient group. The findings indicate that in most cases, women can return to their normal activities; albeit in a different way. The results demonstrate a need for the standardisation of postoperative care among this patient group. The variation in clinical practice could be addressed by the implementation of a PSM to ensure that these resilient women are receiving the support they need at the key time points throughout their recovery.

## Supporting information

S1 File Focus group topic guide. (DOCX)

S2 File Dyad interview schedule. (DOCX)

S3 File Qualitative coding trail. (DOCX)

S4 File SPSS survey dataset. (SAV)
