# Supplementary material for: The musculoskeletal consequences of latissmus dorsi breast reconstruction in women following mastectomy for breast cancer
Source: PLoS One. 2018 Aug 28;13(8):e0202859. doi: 10.1371/journal.pone.0202859 (PMC6112655; doi:10.1371/journal.pone.0202859)
Supplement: S1 File — (DOCX) [file pone.0202859.s001.docx]

**Focus group topic guide**

**Women**

Introduction outlining the research, participant rights and how the focus group will be conducted. Introduce members of the research team, giving participants an opportunity for questions.

**Impact and Extent of Surgery:**

Ice-breaker question to establish a commonality amongst participants:

**Can you please give us a little background information on when you received your cancer diagnosis and when you had your mastectomy and reconstruction surgery?**

**Can you tell us a little about your journey from diagnosis to LD flap surgery, and we are particularly interested in your understanding of the possible impact of the LD flap surgery on your shoulder function before you had it done?**

Prompt: journey from diagnosis to surgery

- Interaction with BCN – when/what was offered?
- Why did you choose LD flap surgery?
- Understanding of consequences of LD flap surgery
- Functional expectations
- Relative importance of factors, e.g. aesthetics and shoulder function
- Importance of timing re decision-making (immediate versus delayed)

**What was the physiotherapy treatment and management that you received either before, during or after the reconstruction surgery?**

- Who got PT and who did not/when did they see the PT in their journey?
- Functional expectations
- Exercises (strengthening, stretching, cardio-vascular, injury prevention)

**Since your breast reconstruction, have you noticed any difference in your involvement in activities of daily living; including, household chores, employment, physical activity, and generally a change in your roles from before surgery to after surgery?**

Prompt: pain, disability, mobility, function, role

**Since your breast reconstruction, have you noticed any difference in your shoulder movement or shoulder function when you compare one side with the other?**

Subsidiary question: how have you managed this?

**Since your breast reconstruction, have you noticed any difference (including aches, pains, stiffness etc.) elsewhere, i.e. aside from your shoulder/s?**

Subsidiary question: how have you managed this/these?

**What do you think are the main benefits of breast reconstruction surgery?**

Prompt: physical, psychological, social

- relative importance of factors, e.g. aesthetics and shoulder function

**What do you think are the main disadvantages of breast reconstruction surgery?**

Prompt: physical, psychological, social

- relative important of factors, e.g. recovery time and function

**Has this reconstruction surgery impacted on any members of your family or friends?**

Prompt: different family members: husband/partner, children (age important?), others?

Subsidiary question: have you tried to control or manage the impact of your surgery on your family and friends?

Subsidiary question: How did you control or manage the impact?

- Who did you seek support from? (role, function, ADL)

**If you had a magic wand, is there anything you would like to change for women coming after you, and I am particularly thinking about whether you would change or add to any advice that you got about shoulder function after the surgery?**

**Is there anything you would like to add?**

**Healthcare Professionals (Breast Care Nurses and Physiotherapists)**

Introduction outlining the research, participant rights and how the focus group will be conducted. Introduce members of the research team, giving participants an opportunity for questions.

Ice-breaker question to establish a commonality amongst participants:

**Can you please give us a little information on your current health care role, and your experience of working with women who have had breast reconstruction using the LD?**

Prompt: specifics of the role:

- decision-making
- advice given to women re preparation for surgery and post-op advice re shoulder etc.
- expectations of women re surgical outcome
- their understanding of the impact of LD flap on the woman
  - extra prompt: MSk impact, function, disability
  - role of exercise

**What do you think are the main benefits of breast reconstruction surgery?**

Prompt: physical, psychological, social

- relative importance of factors, e.g. aesthetics and shoulder function

**What do you think are the main disadvantages of breast reconstruction surgery?**

Prompt: physical, psychological, social

- relative important of factors, e.g. recovery time and function

**What do you think are the main musculoskeletal consequences of breast reconstruction surgery using the LD muscle, if any?**

Prompt: consequences for woman (home, work, hobbies, role)

Prompt: consequences for other family members/inner circle

Prompt: pain, disability, mobility, function, role

**Are you aware of any impact that this surgery has on other members of the family or friends?**

Prompt: different family members: husband/partner, children (age important?), others, how woman manages impact, disclosure to family, impact around decision-making - who is involved in the decision, relative importance of post-op function/role re decision

**Is there anything you would like to add?**
