# Supplementary material for: The musculoskeletal consequences of latissmus dorsi breast reconstruction in women following mastectomy for breast cancer
Source: PLoS One. 2018 Aug 28;13(8):e0202859. doi: 10.1371/journal.pone.0202859 (PMC6112655; doi:10.1371/journal.pone.0202859)
Supplement: S2 File — (DOCX) [file pone.0202859.s002.docx]

**Dyad interview schedule**

**Women**

**Can you please give me a little information on when you received your cancer diagnosis and when you had your mastectomy and reconstruction surgery?**

**Can you tell me a little about your journey from diagnosis to LD flap surgery, and I’m particularly interested in your understanding of the possible impact of the LD flap surgery on your shoulder function before you had it done?**

- Why did you select the LD flap as your method of breast reconstruction?
- Functional expectations of the outcome of surgery?
- Significant others involvement and their understanding of the consequences of surgery?

**So what we’re interested in is function and what we mean by that is everyday activities such as getting in and out of bed, dressing, washing, household chores, exercising, and things like that.**

**While in hospital, can you remember how you were immediately following surgery?**

- Did you receive any physiotherapy treatment?
- Were you given any exercises, who by?
- Was surgery what you had expected?

**Whenever you came home after surgery, how was it?**

- Did you notice any difference in your everyday life e.g. at home? In what way?
- How long did this last?
- What support did you get, who from?
- Did your role change during this time?

**Following LD breast reconstruction was your employment or work impacted at all?**

- Did you return to work?
- Did you return to the same job you had before your reconstruction?
- What was the reasoning for the change?

**Since your breast reconstruction, have you noticed any difference in your involvement in activities of daily living; including, household chores, employment, physical activity, and generally a change in your role from before surgery to after surgery?**

- Are there any on-going challenges or any specific activities or things that have been reduced since surgery e.g. at home/work or physical activity?
- Can you give me any examples?
- Have you changed the way you carry out any tasks?
- Can you give me any examples?
- Is there anything that you would have done that you no longer do e.g. at home/work or physical activity?
- Can you give me any examples?
- Did surgery have an impact on your physical intimate relationship at the time?
- Where do you think you are now in your recovery?
- Is there anything that would have helped?

**During your recovery from LD flap surgery, do you think that any family member or friends were affected?**

- In what way?
- Are they now?
- Were your children affected?
- Do you think the surgery impacted on your wider social circle, for example work?
- What has helped you getting through this experience?

**Thinking back on your experience of LD flap surgery?**

- What were the biggest challenges?
- Were there any positives?
- Is there anything on hindsight you wished you had known beforehand which would have helped?

**Is there anything you feel that we haven’t covered or is there anything that you would like to add?**

**Significant Others**

**Can you please give me a little information on when [name] was diagnosed with breast cancer and had her mastectomy and reconstruction surgery?**

**Can you tell me a little about the journey from diagnosis to LD flap surgery, and I’m particularly interested in your understanding of the possible impact of the LD flap surgery on shoulder function before [name] had it done?**

- Why did she select LD flap surgery as her method of breast reconstruction?
- What was your involvement during the decision making?
- How did you think it was going to affect her?
- Where did you get that information?
- Were you worried about her post-operative function when she decided to undergo reconstructive surgery?
- Where did you look for support?

**So what we’re interested in is function and what we mean by that is everyday activities such as getting in and out of bed, dressing, washing, household chores, exercising, and things like that.**

**While in hospital, can you remember how [name] was immediately following surgery?**

- Did she receive any physiotherapy treatment?
- Was she given any exercises, who by?
- Was surgery what you had expected?

**Whenever [name] came home after surgery, how was it?**

- Did you notice any difference in her everyday life e.g. at home? In what way?
- How long did this last?
- Did her role change during this time?
- Was there any impact on you in the initial post-operative period?
- What support did she get, who from?
- What support did you get?

**Since her breast reconstruction, have you noticed any difference in [name] involvement in activities of daily living; including, household chores, employment, physical activity, and generally a change in her role from before surgery to after surgery?**

- Are there any on-going challenges or any specific activities or things that have been reduced since surgery e.g. at home/work or physical activity?
- Can you give me examples?
- Has she changed the way she carries out any tasks, can you give me any examples?
- Is there anything that she would have done that she no longer does e.g. at home/work or physical activity?
- Can you give me any examples?
- In terms of employment, following surgery did she return to the same job as before?
- What was the reasoning for this change?
- Where do you think she is now in her recovery?
- Is there anything that would have helped?

**During her recovery from LD flap surgery, did you notice any impact on any other family members or friends?**

- In what way?
- Are they impacted now?
- Were her children affected?
- Do you think the surgery impacted on her wider social circle, for example work?

**Looking back on the experience, what’s your understanding of the surgery now?**

- What were the biggest challenges?
- Were there any positives?
- Is there anything on hindsight you wished you had known beforehand which would have helped?

**Is there anything you feel that we haven’t covered or is there anything that you would like to add?**
