# Supplementary material for: The musculoskeletal consequences of latissmus dorsi breast reconstruction in women following mastectomy for breast cancer
Source: PLoS One. 2018 Aug 28;13(8):e0202859. doi: 10.1371/journal.pone.0202859 (PMC6112655; doi:10.1371/journal.pone.0202859)
Supplement: S3 File — (DOCX) [file pone.0202859.s003.docx]

**Qualitative Coding Trail**

**Codes and categories generated from the focus group study: Women’s findings**

| **Codes that informed the categories** | **Categories that informed the themes** | **Themes** | **Overarching theme** |
| --- | --- | --- | --- |
| Preoperative information provided  Postoperative follow-up | Understanding of the impact of surgery and anticipated recovery | Preparation and awareness of the MSk impact of surgery | RESILIENCE |
| Dealing with diagnosis  Recovery expectations | Influence of healthcare professionals |  |  |
| Functional impact  Emotional impact  Body image concerns  Effects of treatment | Challenges associated with surgery/ Changing priorities/ Acceptance of limitations | Coping with the impact of surgery |  |
| Emotional/ Practical support/ Help with personal care and ADL | Aftercare  Follow-up support | Self-managing the effects of surgery |  |
| Delegation/ Compensation/ Avoidance | Adapting to changes following surgery |  |  |

**Codes and categories generated from the focus group study: Healthcare professionals findings**

| **Codes that informed the categories** | **Categories that informed the themes** | **Themes** |
| --- | --- | --- |
| Preoperative PT patient contact varied/ Inconsistent follow-up/ Postoperative PT rehabilitation on referral  BCN involved preoperatively/ BCN short-term surgical follow-up/ MSk problems not within their role | Distinct roles of HCPs  (role of physiotherapist/  role of a BCN) | HCPs perceptions of their role in caring for patients undergoing LD flap surgery |
| Anticipated functional recovery  Impact dependent on pre-morbid level of fitness/ dominance/ circumstances at home  Psychological impact/ Body image concerns/ Personal expectations/ Functional recovery | Varying awareness of MSk impact of surgery  HCPs have differing opinions on the relative importance of the outcomes relating to surgery | HCP perceptions of impact of surgery on women |
| Pathways differ/ Lack of resources  Fragmented multidisciplinary care  Differing perceptions of MSK impact | Recognising challenges | Inconsistencies within service provision |

**Codes and categories generated from the dyad interviews: Women’s findings**

| **Codes that informed the categories** | **Categories that informed the themes** | **Themes** | **Overarching theme** |
| --- | --- | --- | --- |
| Dealing with diagnosis/ household tasks/ personal care/ ADL/ caring responsibilities | Practical support  Emotional support | Significance of support | MANAGING EXPECTATIONS OF SURGERY |
| Sources of support  Role changes/ impact on relationships/ time away from work/ input from HCP | Importance of support networks  Dependency on others |  |  |
| Postoperative pain  Dysfunction/ Weakness  Numbness/ Impact on ADL | Physical implications of surgery  Effects of treatment | Relative importance of outcomes relating to surgery |  |
| Significance of losing a breast/ Scarring/ Body image concerns/ Impact on self-confidence/ Femininity/ A sense of closure from the experience/ Looking the same as everyone else/ Practical aspect of buying clothes | Psychological impact  Aesthetic outcome of surgery  Feelings of normality |  |  |
| Adapting to changes following surgery (delegation/ compensation/ avoidance)  Recognising the importance of functional recovery | Self-management | Responsibility for their own aftercare |  |
| Acceptance of limitations  Redefining normality | Coping strategies |  |  |

**Codes and categories generated from the dyad interviews: Significant other’s findings**

| **Codes that informed the categories** | **Categories that informed the themes** | **Themes** | **Overarching theme** |
| --- | --- | --- | --- |
| Importance of information/ support (family/ HCP) | Preoperative information provided/ Information pursued | Lack of preparedness | MANAGING EXPECTATIONS OF SURGERY |
| Postoperative pain  Functional rehabilitation | Aftercare  Follow-up |  |  |
| Personal care/ Household chores/ Increased caring responsibilities | Managing the home and caretaking | Role adjustments |  |
| Dependency leave  Impact on social life | Return to work  Impact on ADL |  |  |
| Impact on relationship  Psychological impact | Immediate postoperative impact | Impact on daily living |  |
| Effects of treatment (recovery)  Circumstances at home | Long-term implications |  |  |
